# Transmission efficiency of *Plasmodium vivax* at low parasitaemia

**DOI:** 10.1186/s12936-022-04435-9

**Published:** 2023-01-19

**Authors:** Thitiporn Surit, Piyarat Sripoorote, Chalermpon Kumpitak, Chayanut Suansomjit, Nongnuj Maneechai, Liwang Cui, Jetsumon Sattabongkot, Wanlapa Roobsoong, Wang Nguitragool

**Affiliations:** 1 Mahidol Vivax Research Unit, Faculty of Tropical Medicine, Mahidol University, 420/6 Ratchawithi Rd, Ratchathewi, Bangkok, 10400 Thailand; 2 Department of Clinical Tropical Medicine, Faculty of Tropical Medicine, Mahidol University, Bangkok, Thailand; 3 Department of Internal Medicine, Morsani College of Medicine, University of South Florida, Tampa, FL 33612 USA; 4 Department of Molecular Tropical Medicine and Genetics, Faculty of Tropical Medicine, Mahidol University, Bangkok, Thailand

**Keywords:** *Plasmodium vivax*, Transmission, Infectivity, Gametocyte, Malaria, Anopheles, Asymptomatic, Oocyst

## Abstract

**Background:**

*Plasmodium vivax* is responsible for much of malaria outside Africa. Although most *P. vivax* infections in endemic areas are asymptomatic and have low parasite densities, they are considered a potentially important source of transmission. Several studies have demonstrated that asymptomatic *P. vivax* carriers can transmit the parasite to mosquitoes, but the efficiency has not been well quantified. The aim of this study is to determine the relationship between parasite density and mosquito infectivity, particularly at low parasitaemia.

**Methods:**

Membrane feeding assays were performed using serial dilutions of *P. vivax*-infected blood to define the relationship between parasitaemia and mosquito infectivity.

**Results:**

The infection rate (oocyst prevalence) and intensity (oocyst load) were positively correlated with the parasite density in the blood. There was a broad case-to-case variation in parasite infectivity. The geometric mean parasite density yielding a 10% mosquito infection rate was 33 (CI _95_ 9–120) parasites/µl or 4 (CI _95_ 1–17) gametocytes/µl. The geometric mean parasite density yielding a 50% mosquito infection rate was 146 (CI _95_ 36–586) parasites/µl or 13 (CI _95_ 3–49) gametocytes/µl.

**Conclusion:**

This study quantified the ability of *P. vivax* to infect *Anopheles dirus* at over a broad range of parasite densities. It provides important information about parasite infectivity at low parasitaemia common among asymptomatic *P. vivax* carriers.

## Background

Malaria continues to be a global scourge of humans. According to the World Health Organization (WHO), the latest estimate of malaria burden was 229 million cases in 2019, of which 409,000 died [1]. A mosquito-borne disease, malaria is caused by the infection of protozoan parasites of the genus *Plasmodium*. The vast majority of clinical incidences of malaria are due to *Plasmodium falciparum* and *Plasmodium vivax*. In the last two decades, the global malaria reduction effort has led to a significant decrease in the case number, but the progress appeared to have slowed down in the last few years [1]. Because malaria is vector-borne, disease control and elimination strategies rely heavily on vector control measures, such as insecticide-treated nets and indoor residual spraying. Additional means to reduce transmissions such as prompt treatment and reporting, reactive surveillance, and use of the 8-aminoquinoline drug primaquine to inactivate gametocytes have also been adopted by various countries [1–3].

Most *P. vivax* infections in endemic areas are asymptomatic, even in low transmission settings approaching elimination [4–9]. Because asymptomatic carriers can transmit the disease [10–16], they represent a challenge for malaria elimination. At present, the extent to which *P. vivax* carriers contribute to transmission has not been quantified in most settings. Although asymptomatic carriers are much more prevalent than patients, they are less infectious to the mosquitoes due to the lower parasite density in blood. The relative contributions of asymptomatic carriers and patients in a population to malaria transmission depend on the relative prevalence, infectivity, and contact frequency with the mosquito vectors. Few studies have attempted to assess the relative infectivity of asymptomatic carriers and patients [12–17]. Recent studies from Ethiopia and Amazonia predicted that asymptomatic carriers contribute approximately 30–80% of transmission [14, 16]. In mainland Southeast Asia, it was recently found that human asymptomatic infections and mosquito infections arose closely in time after mass drug administration (MDA) of dihydroartemisinin-piperaquine with low dose primaquine, suggesting that asymptomatic carriers be the key drivers of transmission [18].

There remains a gap in understanding of how well *P. vivax* transmits from humans to mosquitoes. Although many mosquito feeding assays have been performed and together provided a valuable dataset of *P. vivax* infectivity [10–15, 19–27], the majority of data were obtained at the clinical level of parasite densities. Far fewer data have been acquired at low-density levels typical of asymptomatic carriers [28]. This study aims to fill this gap by determining the quantitative relationship between *P. vivax* parasite density and mosquito infectivity over a broad range of parasitaemia. Serial dilution of patient blood was used to generate parasite densities at different levels. The membrane feeding assay (MFA) was used with laboratory-reared *Anopheles dirus*, a major vector in Southeast Asia, to measure *P. vivax* transmission.

## Methods

### **Blood collection from*Plasmodium vivax*patients**

*Plasmodium vivax*-infected blood was collected from symptomatic patients who attended malaria clinics in Tak and Ubon Ratchathani provinces of Thailand. The study was approved by the Ethics Committee of Faculty of Tropical Medicine, Mahidol University (MUTM 201 L-040-05). Enrollment was limited to malaria patients who were ≥  18 years old. From each patient, venous blood (20 ml) was collected in a heparinized tube. Blood was immediately placed in a warm box (37 ℃ ± 5 ℃) and transported to a field laboratory or the Mahidol Vivax Research Unit (MVRU) in Bangkok for MFA.

#### Blood preparation and membrane feeding assay

For every *P. vivax*-infected blood sample, *P. vivax* mono-species infection was confirmed by nested PCR as previously described [29, 30]. Blood was washed with pre-warmed serum-free RPMI 1640 medium and centrifuged at 800 × g for 10 min at 37 °C. Packed infected blood was resuspended with an equal volume of naïve human AB serum to 50% haematocrit (hct). The resuspended blood was subjected to 2-fold serial dilution with warmed 50% hct-O cells in human AB serum, generating 13 different levels of parasitaemia. The final volume for each dilution was 600 µl. The diluted blood was kept at 37 °C until MFA. A thick-film slide was prepared at each parasite concentration for microscopic examination by dotting 1 µL of blood at per spot, five spots per side. The remaining blood was used to feed 100 female *An. dirus* mosquitoes using MFA.

### Light microscopic examination of blood smears

Thick blood smears were stained with 10% Giemsa solution for 10 min, rinsed with running tap water, and air dried. Total parasites per 1 µl spot were counted once under a light microscope (LM) for all original and serially diluted blood samples fed to the mosquitoes. Counts were made separately for asexual and sexual stages.

### Nested PCR analysis

To confirm *P. vivax* mono-infection, nested PCR was performed as previously described [29, 30], using genus and species-specific primers against the 18 S ribosomal RNA genes. Briefly, DNA was extracted by QIAamp^®^ DNA Mini Kit (Qiagen, Germany) according to the manufacturer’s instructions. The first round of PCR was performed using the purified DNA as the template with genus-specific primers. The PCR product was then used as the DNA template for a second PCR reaction which used the same forward primer with a species-specific reverse primer. The second PCR was performed in 5 separate reactions, each detecting *P. falciparum*, *P. vivax*, *Plasmodium malariae*, *Plasmodium ovale*, or *Plasmodium knowlesi* DNA. Water was used as the template for the negative controls. The PCR products were visualized on agarose gel after electrophoresis.

### Mosquito rearing

A colony of *An. dirus* was maintained in the insectary of the MVRU. The colony was established in 2011 from the original colony obtained from the Armed Force Research Institute of Medical Sciences, Bangkok, Thailand, as previously described [25]. Briefly, the mosquitoes were reared at 26–27 °C (± 1 °C), 70–80% (± 10%) relative humidity, and with a 12 h day/night cycle. For MFA, female mosquito cartons were placed inside an insulated plastic cooler and ground transported from Bangkok to the field sites. For MFA, female mosquitoes (only 5 to 7 day-old mosquitoes) were used after 6 h sugar starvation. Approximately 100 starved mosquitoes were used for each diluted blood sample.

### Membrane feeding assay (MFA)

To feed 100 female mosquitoes, 400 µL of each diluted blood was added to a water-jacketed glass membrane feeder covered with a Baudruche membrane and maintained at 37 °C with a circulating-water system to prevent the transition of gametocytes to gametes. Mosquitoes were allowed to feed for 30 min. The engorged mosquitoes were maintained with 10% sucrose solution. All engorged-mosquito midguts were dissected on day 7 post feeding, stained with 0.05% mercurochrome, and examined under an LM. Two parameters were determined for each feeding experiment: (i) mosquito infection rate (percent oocyst-positive mosquitoes in all mosquitoes dissected) and (ii) oocyst intensity (the mean oocyst count in all mosquito dissected).

### Data analysis

When available, parasite and gametocyte densities used in the analysis were direct microscopic counts. At low densities, when no parasite was detected under the microscope, the densities were imputed based on the known fold-dilution of the original samples. All imputed values were less than 5 parasites (or gametocytes) per µl. To determine the parasite (or gametocyte) densities yielding 10% and 50% oocyst prevalence, the rising phase of each dose response (from 0% up to 85% infection rate) was fit to the Hill’s equation using *Quest Graph IC50 Calculator* [31] with two free parameters, the Hill coefficient and the half-maximal concentration; the minimum and maximum infection rates were set to 0% and 100%, respectively.

## Results

### General characteristics of P. vivax isolates

Eight *P. vivax*-infected blood samples were obtained from different malaria patients who visited a health facility in Thailand in 2016–2017. All infections were confirmed to be mono-species by nested PCR [30]. The infection characteristics are summarized in Table 1. The geometric mean parasite density was 1328 (CI_95_ 593–2973) parasites/µL. The geometric mean gametocyte density was 153 (CI_95_ 92–254) gametocytes/µL. The mean female/male ratio was 4.4 (CI_95_ 1.6–7.2).

**Table 1:** Characteristics of *P. vivax* isolates used in membrane feeding assays

*P. vivax* Patient	Parasite density /µl	Gametocyte density /µl			F:M ratio	Infection rate before dilution (%)
		Total	Female	Male		
VTTY121	325	45	28	17	1.7	90
VUBR24	2479	363	281	82	3.4	90
VUBR25	576	95	74	21	3.6	50
VUBR28	1890	149	113	36	3.1	80
VUBR33	3399	256	209	47	4.5	89
VUNL24	5285	167	155	12	12.6	37
VUNL25	234	ND^a^	ND^a^	ND^a^	-	58
VUNL28	2626	200	134	67	2.0	95

^a^ not detected

- Value cannot be determined because no gametocyte was observed

### Relationship between parasite density and mosquito infection

**Fig. 1:**
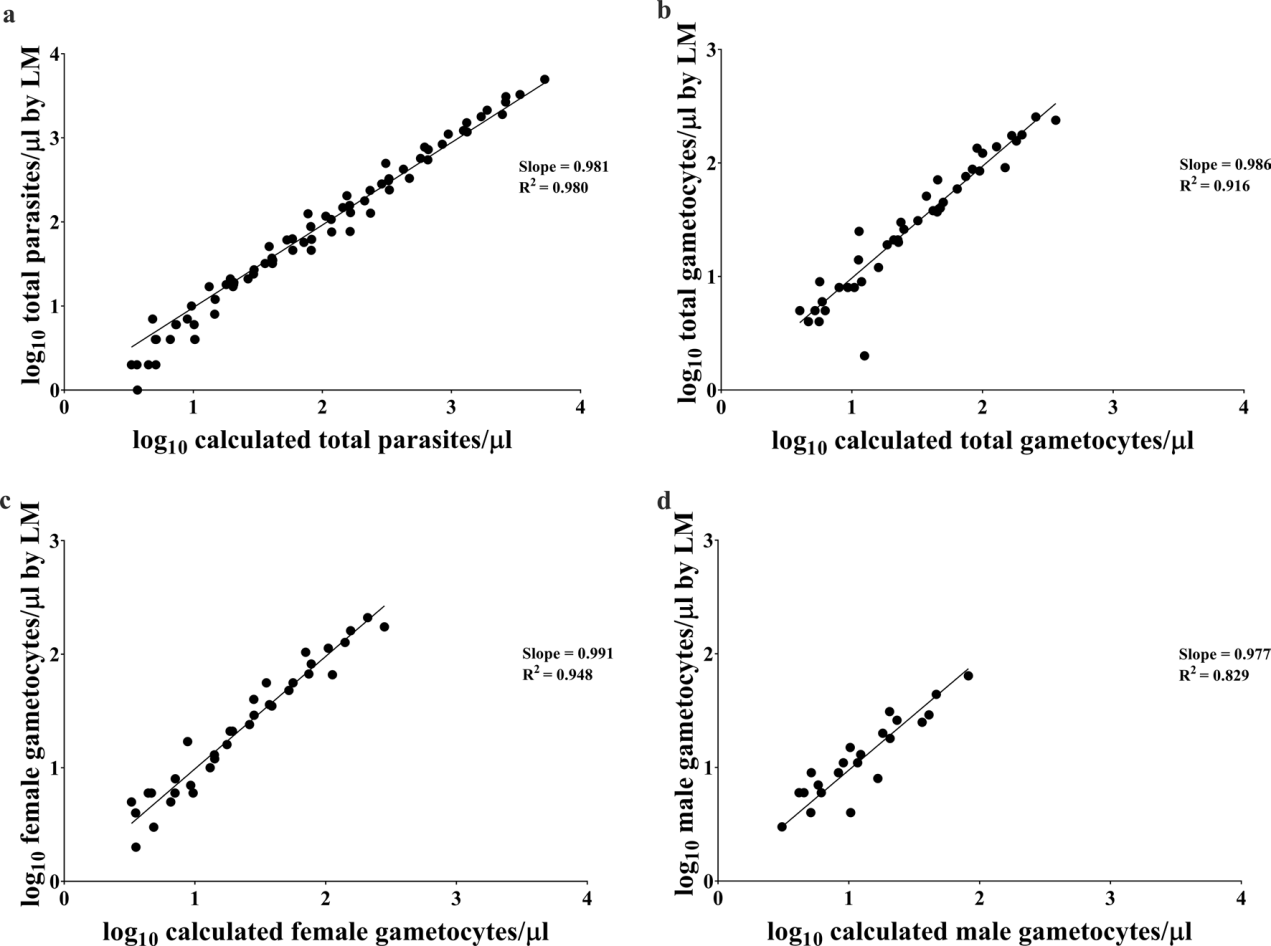
*Plasmodium vivax* densities in serially diluted blood samples. Shown are the relationships between actual LM counts and calculated parasite densities based on the dilution factors for **a** total blood-stage parasites, **b** total gametocytes, **c** female gametocytes and **d** male gametocytes. For each plot, data from all 8 cases were combined. Only data with calculated densities above 3 per microliter were included. Trendlines represent the best linear fits with zero crossing on the log-log scale

Twelve parasite densities were obtained for each parasite isolate in addition to the original parasite density. The lowest data point represents a 4096-fold reduction of parasite density relative to the original. The parasite density of each diluted blood sample was re-evaluated by LM to ensure that no gross error was introduced by serial dilution. Figure 1 demonstrates the consistency between the LM counts and the calculated parasite densities based on known dilution factors. Actual counts were used in all analysis, except when no parasite was detected, in which case the imputed parasite density value was used.

**Fig. 2:**
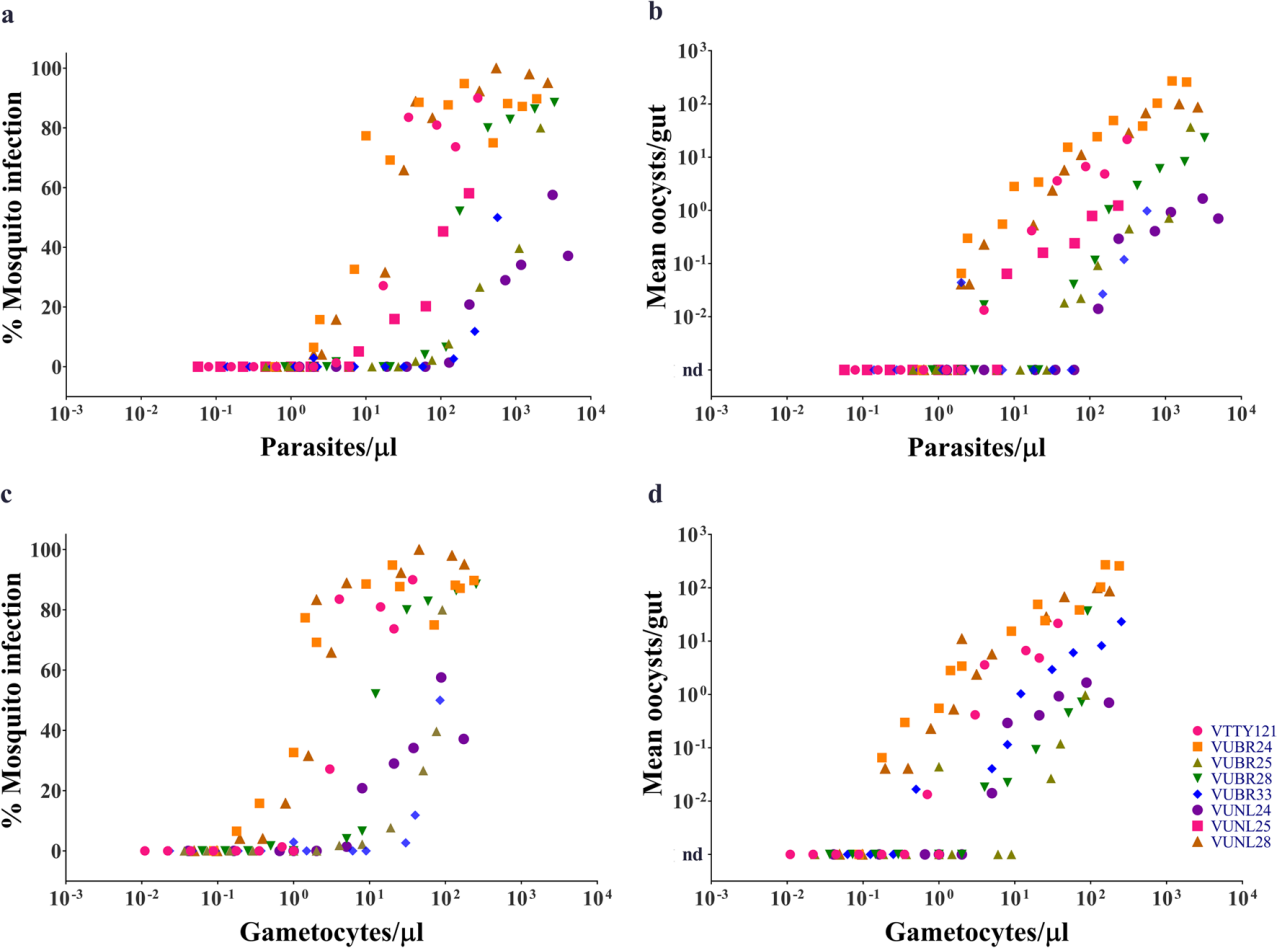
Mosquito infection as a function of parasite or gametocyte densities. **a** mosquito infection rate vs. total parasitaemia, **b** the oocyst intensity vs. total parasitaemia, **c** mosquito infection rate vs. total gametocytaemia and **d** the oocyst intensity vs. total gametocytaemia

In all cases, the MFA showed that both the mosquito infection rate and the oocyst intensity declined as the infected blood was diluted (Fig. 2). In 5/8 cases, a complete or nearly complete dose response was observed. In these cases, the infection rate was over 80% at the highest parasite density (no dilution) and decreased to 0% after the blood was sufficiently diluted. In the three other cases, the infection rate at the highest parasite density was 37–58% resulting in a partial dose response. The lowest infective parasite density (IP_L_), i.e. the parasite density causing infection in at least one mosquito, ranged from 2 to 129 parasites/µl. The lowest infective gametocyte density (IG_L_) ranged from 0.2 to 5 gametocytes/µl (Table 2).

**Table 2:** Lowest infective parasite and gametocyte densities

*P. vivax* ID	IP_L_^*^ (parasite/µl)	IG_L_^**^ (gametocytes/µl)	Mosquito infection frequency at IP_L_ (infected / dissected)
VTTY121	4	0.7	1 / 75
VUBR24	2	0.2	3 / 46
VUBR25	2	1	2 / 68
VUBR28	46	4	1 / 55
VUBR33	4	0.5	1 / 60
VUNL24	129	5	1 / 71
VUNL25	8	–	4 / 78
VUNL28	3	0.2	2 / 49
Geometric mean (CI _95_ )	7 (2–21)	0.8 (0.3–2)	

* IP_L_: lowest infective parasitaemia

** IG_L_: lowest infective gametocytaemia

– Value cannot be determined because no gametocyte was observed

The dose response of the mosquito infection rate was further analyzed by non-linear regression. For each parasite isolate, the characteristic parasitaemia resulting in 10% and 50% mosquito infection, referred to as IP_10_ and IP_50_, respectively, were determined by fitting the dose response to Hill’s equation and solving the best-fit equation for the 10% and 50% infection rates respectively. The infective gametocytaemias (IG_10_ and IG_50_) were similarly determined. Table 3 displays these parameters for each parasite isolates. The geometric means of IP_10_ and IG_10_ were 33 (CI_95%_ 9–120) parasites/µl and 4 (CI_95%_ 1–17) gametocytes/ µl, respectively. The geometric means of IP_50_ and IG_50_ were 146 (CI_95% _ 36–586) parasites/µl and 13 (CI_95%  _3–49) gametocytes/µl, respectively. Thus, on average, 33 parasites/µl (or 4 gametocytes/µl) was required to achieve 10% infection in *An. dirus*. Similarly, 146 parasites/µl (or 13 gametocytes/µl) was required to infect 50% of the mosquitoes.

**Table 3:** Characteristic parasitaemia and gametocytaemia

*P. vivax* ID	IP_10_^*^ (parasites/µl)	IP_50_^**^ (parasites/µl)	IG_10_^***^ (gametocytes/µl)	IG_50_^****^ (gametocytes/µl)
VTTY121	12	23	3	3
VUBR24	2	8	0.3	1
VUBR25	263	569	39	85
VUBR28	176	1045	40	73
VUBR33	80	217	5	15
VUNL24	186	2175	7	64
VUNL25	20	162	–	–
VUNL28	5	24	0.7	2
Geomean CI_95_	33 9–120	146 36–586	4 1–17	13 3–49

* IP_10_: parasitaemia at 10% mosquito infection rate

** IP_50_: parasitaemia at 50% mosquito infection rate

*** IG_10_: gametocytaemia at 10% mosquito infection rate

**** IG_50_: gametocytaemia at 50% mosquito infection rate

– Value cannot be determined because no gametocyte was observed

### Relationship between the oocyst density and mosquito infection rate

**Fig. 3:**
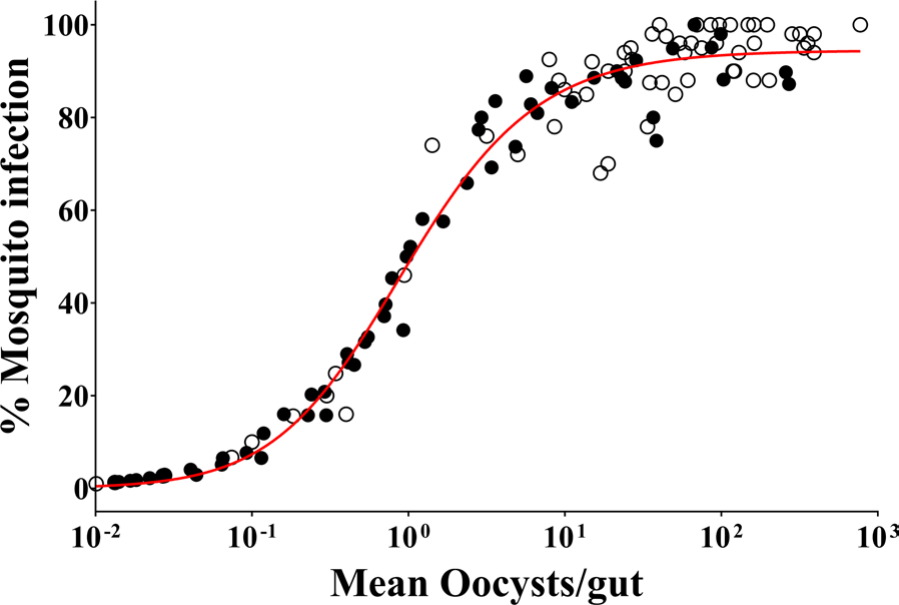
Relationship between oocyst density and mosquito infection rate. Black circles represent values from individual feeding experiments in this study. White circles represent values from individual feeding experiments with AB serum replacement from a previous study [25]. The solid red line is the best fit by Hill’s equation

The data from the feeding experiments of the 8 isolates demonstrated a well-defined and robust relationship between the mosquito infection rate and the oocyst intensity (Fig. 3). The trend (filled symbols) increased monotonically and plateaued near the 100% infection rate. This relationship is nearly identical to the previously published one (open symbol) [25] despite the two studies being conducted several years apart. Therefore, the relationship between the mosquito infection rate and the oocyst intensity is robust; one parameter can reasonably predict the other.

## Discussion

The relationship between *P. vivax* parasite density in blood and mosquito infectivity is important for estimating the potential of each infected individual to transmission. Several studies have examined this relationship [12, 14, 20, 21, 23–25], but the results are variable, presumably reflecting biological differences between different mosquito species and/or parasite isolates as well as methodological differences in the feeding assays [28]. For example, in a study from Ethiopia using *Anopheles arabiensis* and *Anopheles pharoensis* [23], the infection rate by MFA was found to increase with gametocyte density and plateaued at about 30–40%. In the same study, the gametocyte density of 50–100 gametocytes/µl was needed to attain the sizable infection rate of 20%. A separate study using *An. arabiensis* [24] also found that similarly high gametocyte densities were needed to achieve this level of infection. In contrast, in a study using *An. dirus* [25], only a few *P. vivax* gametocytes/µl were sufficient to result in a 20% infection rate. In this study, MFAs were performed using serially diluted patient blood with the specific aim of generating a dataset with a balanced parasite density distribution. It was found that the lowest parasite density that gave rise to mosquito infection ranged from 2 to 129 parasites/µl, or from undetectable to 5 gametocytes/µl. While these density values provide a glimpse of transmission potential at low parasite densities, they have limited utility. This is because the values of IP_L_ and IG_L_ depend on the absolute number of mosquitoes examined for infection. As more mosquitoes are examined, IP_L_ and IG_L_ will tend to be lower due to the increased oocyst detection sensitivity. Because of this, it is preferable to characterize the mosquito infectivity by characteristic concentrations like IP_10_ or IG_10_. In this study, the geometric mean of IP_10_ was 33 (CI_95%_ 9–120) parasites/µl and the geometric mean of IG_10_ was 4 (CI_95%_ 1–17) gametocytes/µl. The 95% confidence interval was broad, reflecting high variation across different feeding experiments. Of note, the value of IP_10_ is similar to the value predicted by the trendline in a previous study [25], which used 94 independent *P. vivax* isolates, each at a single parasite density. Thus, the serial dilution MFA provided comparable mosquito infection data with far fewer parasite isolates.

The value of IP_10_ of 33 parasites/µl suggests that many asymptomatic *P. vivax* carriers have non-negligible potential to transmit. According to the estimated density distribution of *P. vivax* in Southeast Asia [32], 22% of asymptomatic carriers are predicted to have parasite density higher than 33 parasites/µl. With the following set of simple assumptions: (a) *An. dirus* as the sole vector, (b) asymptomatic carriers representing 95% of all *P. vivax* infections at a given time, (c) 22% of carriers transmitting at the 10% infection rate (or higher), (d) carriers receiving twice more mosquito bites than patients based on the relative outdoor/indoor biting rates [33], and (e) all *P. vivax* patients transmitting at the average 80% infection rate [25], the contribution of asymptomatic carriers to onward transmission would be similar to that of patients, if not higher. Thus, in Southeast Asia, asymptomatic carriers are likely a critical infectious reservoir. Consistently, in a previous MDA study, it was found that *P. vivax* asymptomatic infections in humans and wild mosquitoes arose closely in time after the blood stage clearance by the MDA, suggesting a close relationship between the two [18].

This study also has a few limitations. Using serum replacement and serial dilution in MFA imposed two unnatural conditions that may impact the interpretation of the findings. Firstly, MFA may not fully reflect natural transmission through skin feeding. Although studies have suggested that MFA is an acceptable surrogate for natural feeding [19, 34, 35], these studies were conducted at high parasitaemia using patient blood. There is currently no evidence for, or against, the use of MFA for determining transmission at very low *P. vivax* density. It is plausible that gametocytes are sequestered in the skin to promote their uptake during blood feeding [36, 37], in which case the true potential of *P. vivax* transmission would have been underestimated by MFA. On the other hand, other unknown factors may also impede transmission from the skin in which case MFA would have overestimated the transmission. Secondly, serially diluting blood in naive AB serum with O red cells removed the immunological factors in the original blood which can influence transmission. Plasma components such as naturally acquired antibodies have been shown to modulate parasite transmission, either by inhibiting it [38, 39] or promoting it [40]. Other soluble mediators including cytokines could also interfere [35, 41–43]. With serum replacement, the experiments in this study did not capture these effects. However, in transmission settings where malaria has become low for several years, the effect of antibodies is likely to be small. This is because naturally acquired transmission-blocking immunity had a short duration, declining significantly within months without boosting [39, 44]. Consistent with this, the infectivity dose response in a previous study conducted in a low transmission setting of Thailand was not significantly affected by plasma replacement [25].

## Conclusion

The present study quantified the sexual transmission of *P. vivax* at different parasite dilutions, encompassing the parasitaemia range found in asymptomatic blood-stage parasite carriers. The findings provide critical information for estimating the contribution of asymptomatic infections in malaria transmission. The data suggest that the asymptomatic reservoir is an important source of transmission.

## Data Availability

The datasets used and/or analysed during the current study are available from the corresponding author on reasonable request.

## Abbreviations

LM: Light microscopy
MDA: Mass drug administration
MFA: Membrane feeding assay
IP: Infective parasitaemia
IG: Infective gametocytaemia

## References

[1] World malaria report (2020). 20 years of global progress and challenges.

[2] Lertpiriyasuwat C, Sudathip P, Kitchakarn S, Areechokchai D, Naowarat S, Shah JA (2021). Implementation and success factors from Thailand’s 1–3-7 surveillance strategy for malaria elimination. Malar J.

[3] Cao J, Sturrock HJ, Cotter C, Zhou S, Zhou H, Liu Y (2014). Communicating and monitoring surveillance and response activities for malaria elimination: China’s “1-3-7” strategy. PLoS Med.

[4] Vasquez-Jimenez JM, Arevalo-Herrera M, Henao-Giraldo J, Molina-Gomez K, Arce-Plata M, Vallejo AF (2016). Consistent prevalence of asymptomatic infections in malaria endemic populations in Colombia over time. Malar J.

[5] Waltmann A, Darcy AW, Harris I, Koepfli C, Lodo J, Vahi V (2015). High rates of asymptomatic, sub-microscopic *Plasmodium vivax* infection and disappearing *Plasmodium falciparum* malaria in an area of low transmission in Solomon Islands. PLoS Negl Trop Dis.

[6] Nguitragool W, Mueller I, Kumpitak C, Saeseu T, Bantuchai S, Yorsaeng R (2017). Very high carriage of gametocytes in asymptomatic low-density *Plasmodium falciparum* and P. vivax infections in western Thailand. Parasit Vectors.

[7] Shimizu S, Chotirat S, Dokkulab N, Hongchad I, Khowsroy K, Kiattibutr K (2020). Malaria cross-sectional surveys identified asymptomatic infections of *Plasmodium falciparum*, *Plasmodium vivax* and *Plasmodium knowlesi* in Surat Thani, a southern province of Thailand. Int J Infect Dis.

[8] Hailemeskel E, Tebeje SK, Behaksra SW, Shumie G, Shitaye G, Keffale M (2021). The epidemiology and detectability of asymptomatic *Plasmodium vivax* and *Plasmodium falciparum* infections in low, moderate and high transmission settings in Ethiopia. Malar J.

[9] Sattabongkot J, Suansomjit C, Nguitragool W, Sirichaisinthop J, Warit S, Tiensuwan M (2018). Prevalence of asymptomatic *Plasmodium* infections with sub-microscopic parasite densities in the northwestern border of Thailand: a potential threat to malaria elimination. Malar J.

[10] Coleman RE, Kumpitak C, Ponlawat A, Maneechai N, Phunkitchar V, Rachapaew N (2004). Infectivity of asymptomatic *Plasmodium*-infected human populations to *Anopheles dirus* mosquitoes in western Thailand. J Med Entomol.

[11] Vantaux A, Samreth R, Piv E, Khim N, Kim S, Berne L (2018). Contribution to malaria transmission of symptomatic and asymptomatic parasite carriers in Cambodia. J Infect Dis.

[12] Martins-Campos KM, Kuehn A, Almeida A, Duarte APM, Sampaio VS, Rodriguez IC (2018). Infection of *Anopheles aquasalis* from symptomatic and asymptomatic *Plasmodium vivax* infections in Manaus, western brazilian Amazon. Parasit Vectors.

[13] Vallejo AF, Garcia J, Amado-Garavito AB, Arevalo-Herrera M, Herrera S (2016). *Plasmodium vivax* gametocyte infectivity in sub-microscopic infections. Malar J.

[14] Tadesse FG, Slater HC, Chali W, Teelen K, Lanke K, Belachew M (2018). The relative contribution of symptomatic and asymptomatic *Plasmodium vivax* and *Plasmodium falciparum* infections to the infectious reservoir in a low-endemic setting in Ethiopia. Clin Infect Dis.

[15] Alves FP, Gil LH, Marrelli MT, Ribolla PE, Camargo EP, Da Silva LH (2005). Asymptomatic carriers of *Plasmodium* spp. as infection source for malaria vector mosquitoes in the brazilian Amazon. J Med Entomol.

[16] Ferreira M, Corder R, Johansen I, Kattenberg J, Moreno M, Rosas-Aguirre A (2022). Relative contribution of low-density and asymptomatic infections to *Plasmodium vivax* transmission in the Amazon: pooled analysis of individual participant data from population-based cross-sectional surveys. Lancet Reg Health Am.

[17] Almeida GG, Costa PAC, Araujo MDS, Gomes GR, Carvalho AF, Figueiredo MM (2021). Asymptomatic *Plasmodium vivax* malaria in the brazilian Amazon: submicroscopic parasitemic blood infects *Nyssorhynchus darlingi*. PLoS Negl Trop Dis.

[18] Chaumeau V, Kajeechiwa L, Fustec B, Landier J, Naw Nyo S, Nay Hsel S (2019). Contribution of asymptomatic *Plasmodium* infections to the transmission of malaria in Kayin State, Myanmar. J Infect Dis.

[19] Sattabongkot J, Maneechai N, Rosenberg R (1991). *Plasmodium vivax*: gametocyte infectivity of naturally infected thai adults. Parasitology.

[20] Bharti AR, Chuquiyauri R, Brouwer KC, Stancil J, Lin J, Llanos-Cuentas A (2006). Experimental infection of the neotropical malaria vector *Anopheles darlingi* by human patient-derived *Plasmodium vivax* in the peruvian Amazon. Am J Trop Med Hyg.

[21] Zhu G, Xia H, Zhou H, Li J, Lu F, Liu Y (2013). Susceptibility of *Anopheles sinensis* to *Plasmodium vivax* in malarial outbreak areas of central China. Parasit Vectors.

[22] Moreno M, Tong C, Guzman M, Chuquiyauri R, Llanos-Cuentas A, Rodriguez H (2014). Infection of laboratory-colonized *Anopheles darlingi* mosquitoes by *Plasmodium vivax*. Am J Trop Med Hyg.

[23] Abduselam N, Zeynudin A, Berens-Riha N, Seyoum D, Pritsch M, Tibebu H (2016). Similar trends of susceptibility in *Anopheles arabiensis* and *Anopheles pharoensis* to *Plasmodium vivax* infection in Ethiopia. Parasit Vectors.

[24] Chali W, Ashine T, Hailemeskel E, Gashaw A, Tafesse T, Lanke K (2020). Comparison of infectivity of *Plasmodium vivax* to wild-caught and laboratory-adapted (colonized) *Anopheles arabiensis* mosquitoes in Ethiopia. Parasit Vectors.

[25] Kiattibutr K, Roobsoong W, Sriwichai P, Saeseu T, Rachaphaew N, Suansomjit C (2017). Infectivity of symptomatic and asymptomatic *Plasmodium vivax* infections to a southeast asian vector, *Anopheles dirus*. Int J Parasitol.

[26] Timinao L, Vinit R, Katusele M, Koleala T, Nate E, Czeher C (2021). Infectivity of symptomatic malaria patients to *Anopheles farauti* colony mosquitoes in Papua New Guinea. Front Cell Infect Microbiol.

[27] Araujo MDS, Andrade AO, Dos Santos NAC, Castro RB, Pereira DB, Rodrigues MMS (2020). First observation of experimental *Plasmodium vivax* infection of three malaria vectors from the brazilian Amazon. Vector Borne Zoonotic Dis.

[28] Bantuchai S, Imad H, Nguitragool W (2021). *Plasmodium vivax* gametocytes and transmission. Parasitol Int.

[29] Ngernna S, Rachaphaew N, Thammapalo S, Prikchoo P, Kaewnah O, Manopwisedjaroen K (2019). Case series of human *Plasmodium knowlesi* infection on the southern border of Thailand. Am J Trop Med Hyg.

[30] Yorsaeng R, Saeseu T, Chotivanich K, Felger I, Wampfler R, Cui L (2019). Indigenous *Plasmodium malariae* infection in an endemic population at the Thai-Myanmar border. Am J Trop Med Hyg.

[31] Quest, Graph™. EC50 Calculator (v.1). https://www.aatbio.com/tools/ec50-calculator-v1%20.

[32] Imwong M, Stepniewska K, Tripura R, Peto TJ, Lwin KM, Vihokhern B (2016). Numerical distributions of parasite densities during asymptomatic malaria. J Infect Dis.

[33] Tananchai C, Pattanakul M, Nararak J, Sinou V, Manguin S, Chareonviriyaphap T (2019). Diversity and biting patterns of *Anopheles* species in a malaria endemic area, Umphang Valley, Tak Province, Western Thailand. Acta Trop.

[34] Sattabongkot J, Maneechai N, Phunkitchar V, Eikarat N, Khuntirat B, Sirichaisinthop J (2003). Comparison of artificial membrane feeding with direct skin feeding to estimate the infectiousness of *Plasmodium vivax* gametocyte carriers to mosquitoes. Am J Trop Med Hyg.

[35] Vallejo AF, Rubiano K, Amado A, Krystosik AR, Herrera S, Arevalo-Herrera M (2016). Optimization of a membrane feeding assay for *Plasmodium vivax* infection in *Anopheles albimanus*. PLoS Negl Trop Dis.

[36] Gaillard FO, Boudin C, Chau NP, Robert V, Pichon G (2003). Togetherness among *Plasmodium falciparum* gametocytes: interpretation through simulation and consequences for malaria transmission. Parasitology.

[37] Lawniczak MK, Eckhoff PA (2016). A computational lens for sexual-stage transmission, reproduction, fitness and kinetics in *Plasmodium falciparum*. Malar J.

[38] Mendis KN, Munesinghe YD, de Silva YN, Keragalla I, Carter R (1987). Malaria transmission-blocking immunity induced by natural infections of *Plasmodium vivax* in humans. Infect Immun.

[39] Arevalo-Herrera M, Solarte Y, Rocha L, Alvarez D, Beier JC, Herrera S (2011). Characterization of *Plasmodium vivax* transmission-blocking activity in low to moderate malaria transmission settings of the colombian Pacific coast. Am J Trop Med Hyg.

[40] Peiris JS, Premawansa S, Ranawaka MB, Udagama PV, Munasinghe YD, Nanayakkara MV (1988). Monoclonal and polyclonal antibodies both block and enhance transmission of human *Plasmodium vivax* malaria. Am J Trop Med Hyg.

[41] Abeles SR, Chuquiyauri R, Tong C, Vinetz JM (2013). Human host-derived cytokines associated with *Plasmodium vivax* transmission from acute malaria patients to *Anopheles darlingi* mosquitoes in the peruvian Amazon. Am J Trop Med Hyg.

[42] Karunaweera ND, Carter R, Grau GE, Kwiatkowski D, Del Giudice G, Mendis KN (1992). Tumour necrosis factor-dependent parasite-killing effects during paroxysms in non-immune *Plasmodium vivax* malaria patients. Clin Exp Immunol.

[43] Naotunne TS, Karunaweera ND, Mendis KN, Carter R (1993). Cytokine-mediated inactivation of malarial gametocytes is dependent on the presence of white blood cells and involves reactive nitrogen intermediates. Immunology.

[44] Ranawaka MB, Munesinghe YD, de Silva DM, Carter R, Mendis KN (1988). Boosting of transmission-blocking immunity during natural *Plasmodium vivax* infections in humans depends upon frequent reinfection. Infect Immun.

